# Bile Duct Injuries Following Laparoscopic Cholecystectomy: A Clinical Study

**DOI:** 10.4103/1319-3767.61236

**Published:** 2010-04

**Authors:** Waheeb R. Al-Kubati

**Affiliations:** Department of Surgery, Althowra Modern General Hospital, Sana'a, Yemen

**Keywords:** Laparoscopic, open, cholecystectomy, common bile duct, chronic cholecystitis, complications

## Abstract

**Background/Aim:**

This study aimed at assessing the outcome of laparoscopic cholecystectomy (LC) by determining the frequency of complications, especially of bile duct injuries.

**Materials and Methods:**

The case files of all patients undergoing laparoscopic cholecystectomy between 2002 and 2006 (inclusive) at King Hussein Medical Center (KHMC) were retrospectively analyzed. We evaluated the data according to outcome measures, such as bile duct injury, morbidity, mortality and numbers of patients whose resections had to be converted from laparoscopic to open.

**Results:**

During the four years (January 2002 and December 2006), 336 patients underwent LC for chronic cholecystitis (CC), of whom 22 (6.5%) developed complications. Among those who developed complications, two patients had major bile duct injuries (0.4%); 43 other patients (12.8%) had planned laparoscopic operations converted to open cholecystectomy intra-operatively. None of the patients in this study died as a result of LC.

**Conclusion:**

Bile duct injury is a major complication of LC. Anatomical anomalies, local pathology, and poor surgical techniques are the main factors responsible. The two patients who had severe common bile duct injury in this study had major anatomical anomalies that were only recognized during surgery.

Operative bile duct injury is one of the serious complications of hepatobiliary surgery. With the introduction of laparoscopic cholecystectomy (LC) there has been an increase in the incidence of such injuries.[1–4] This study was designed to help surgeons in their attempts to prevent undesirable outcomes.

## MATERIALS AND METHODS

In this study the files of 336 patients who underwent LC at the King Hussein Medical Center (KHMC) over four years between January 2002 and December 2006 were collected and analyzed. The cases were evenly distributed over the four years. The patients’ ages ranged from 20 to 60 years; 84 were males and 252 were females. Operating surgeons were divided into two groups; consultants and junior surgeons (those who worked under supervision and were in the residency program). All patients had chronic cholecystitis (CC) and had been symptomatic for at least six months. In all patients the presence of gall stones had been established by ultrasonography. Standard Ethicon^®^ instruments were used for all laparoscopic procedures. Complications were divided into major [extrahepatic common bile duct (CBD) injury which required hepatojejunostomy] and minor [wound infection, minor biliary leak, transient jaundice and ileus). In 45 of these patients a decision had been made to convert the operation intra-operatively from laparoscopic to open, for reasons that include previously undetected presence of adhesions and a difficult anatomy. In two of the 45 patients there were major iatrogenic extrahepatic biliary duct injuries which required immediate hepatojejunostomy: One was a 25-year-old male patient and the other was a 30-year-old female. No long-term follow-up was available to assess late complications.

## RESULTS

Of the 336 patients, 252 (75%) were women and 84 (25%) were men [Table 1].

**Table 1:** Sex distribution and conversion rates in 336 planned laparoscopic cholecystectomies

**Sex**	**No. of cases (%)**	
	
	**Laparoscopic cholecystectomy (%)**	**Converted to open cholecystectomy (%)**
Males	252 (75)	5 (1.5)
Females	84 (25)	40 (11.9)
Total	336 (100)	45 (13.4)

Complications occurred in 22 (6.5%) patients; two (9.1%) had major complications and 20 (88.9%) patients had minor complications [Table 2], the latter included twelve minor wound infections (54.5% - all female), biliary leaks in 13.7% (three patients; one male and two females), all of whom were treated conservatively; transient jaundice in (13.7%) (three patients, all females); and ileus in (18.2%) (four patients) [Table 2]. All major complications occurred in the age-group of 20–30 years, whereas minor complications occurred mainly in the older patients (>41 years) [Table 3]. Minor complications occurred mainly in 16 male patients (72.7%) and only 6 female patients (27.3%). It was not possible to include patients' body mass index, as the data was unavailable. Planned laparoscopic procedures were converted intra-operatively to open procedures in 45 patients (13.4%); two of whom (one male and one female) had open cholecystectomies with CBD repair [Table 1]. All cases of conversion and major complications and (72.7%) (*n*=16) of minor complications, occurred at the hands of consultants. Only (27.3%) (*n*=6) of minor complications occurred at the hands of junior surgeons [Table 2]. There were no mortalities in our study.

**Table 2:** Type and rate of complications vs surgeon group in 22 of 336 laparoscopic cholecystectomies

**Type of complication**	**No. of cases (%)**			
	
	**Senior surgeon**		**Junior surgeon**	
		
	M (%)	F (%)	M (%)	F (%)
Major complication	1 (4.5)	1 (4.5)	0 (0)	0 (0)
Minor complication	4 (18.2)	10 (45.5)	2 (9.1)	4 (18.2)
Wound infection	0 (0.0)	8 (36.)	0 (0)	2 (9.1)
Biliary leak	1 (4.5)	1 (4.5)	0 (0)	1 (4.5)
Transient jaundice	1 (4.5)	0 (0)	1 (4.5)	1 (4.5)
Ileus	3 (13.7)	0 (0.0)	1 (4.5)	0 (0)
Total complications	5 (22.7)	11 (50)	2 (9.1)	4 (18.2)
Total complications by surgeon	16 (72.2)		6 (27.8)			

**Table 3:** Age distribution vs type of complications among 22 out of 336 laparoscopic cholecystectomies

	**10–20 years**		**21–30 years**		**31–40 years**		**41–50 years**		**51–60 years**		**>60 years**	
						
	**M (%)**	**F (%)**	**M (%)**	**F (%)**	**M (%)**	**F (%)**	**M (%)**	**F (%)**	**M (%)**	**F (%)**	**M (%)**	**F (%)**
Major complications	0 (0)	0 (0)	1 (4.5)	1 (4.5)	0 (0)	0 (0)	0 (0)	0 (0)	0 (0)	0 (0)	0 (0)	0 (0)
Minor complications	0 (0)	0 (0)	0 (0)	0 (0)	0 (0)	0 (0)	0 (0)	4 (9)	9 (42)	2 (9)	4 (18)	3 (14)
Wound infection	0 (0)	0 (0)	0 (0)	0 (0)	0 (0)	0 (0)	0 (0)	(36.4)	0 (0)	2 (9)	0 (0)	2 (9)
Biliary leak	0 (0)	0 (0)	0 (0)	0 (0)	0 (0)	0 (0)	1 (4)	0 (0)	0 (0)	1 (4.5)	0 (0)	1 (4.5)
Transient jaundice	0 (0)	0 (0)	0 (0)	0 (0)	0 (0)	0 (0)	1 (4)	1 (4.5)	0 (0)	1 (4.5)	0 (0)	0 (0)
Ileus	0 (0)	0 (0)	0 (0)	0 (0)	0 (0)	0 (0)	3 (14)	0 (0)	1 (4.5)	0 (0)	0 (0)	0 (0)
Total number	0 (0)	0 (0)	1 (4.5)	1 (4.5)	0 (0)	0 (0)	4 (9)	9 (42)	2 (9)	4 (18.2)	0 (0)	3 (14)

## DISCUSSION

Carl Langenbuch performed the first open cholecystectomy in 1882.[5] As surgeons gained more experience and open biliary operations became standardized, the incidence of bile duct injuries reduced to approximately 0.125%.[6,7] Open cholecystectomy remained the gold standard for treatment of cholelithiasis until the late 1980s when LC was introduced.[8] It gained widespread acceptance and became the new gold standard for the management of gall stone diseases. During the surgical learning curve for this new technique there was an initial rise in the reports of bile duct injuries,[1] resulting mainly from the surgeons’ inexperience and misinterpretation of anatomy. Though the reported figures of operative bile duct injuries are much lower than the actual incidence, a recent audit of 1522 LCs performed in Thailand revealed a bile duct injury rate of 0.59%,[3] i.e., about four times the incidence reported for open cholecystectomy; this injury rate is similar to that found in our study (0.6%). In Jordan in 2001, of 791 patients with CC and 207 with acute cholecystitis (AC) who underwent LC, extrahepatic bile duct injuries were reported in only three cases.[6] After 1995, a median incidence rate of 0.3% was documented in data from both retrospective and prospective series.[6,7] The single most important factor responsible for bile duct injuries is misinterpretation of the patient's anatomy. Compared to the open operation, injuries sustained during LC are more often severe (e.g., excision of a segment of the CBD) and generally extend to higher levels. The majority (70–85%) of these injuries are not recognized during the operation; however, both cases of major complications in our series were recognized during surgery and both were iatrogenic injuries as a result of anatomical anomalies. Combined bile duct and hepatic arterial (right hepatic artery or common hepatic artery) injuries carry a particularly bad prognosis, with higher postoperative morbidity and mortality and poorer outcomes after remedial surgery.[9] Bile duct injuries, substantially increase the economic burden on the patient, hospital, and community. Repair of a bile duct injury costs 4.5 to 26 times the cost of an uncomplicated LC and carries the risk of complications and even death.[9] Though the initial spike in the incidence of complications settled down as surgeons became more experienced, reports of major bile duct injuries, even in the hands of senior surgeons, continue to surface, suggesting that bile duct injuries following cholecystectomy will always remain a significant problem. However, early recognition (during operation or in the early post-operative period) improves the outcome and reduces the costs.[10] In our study, all major complications occurred at the hands of consultants and were recognized intra-operatively; only 20% of minor complications occurred at the hands of junior surgeons. The two cases of major complications in our study were due to anatomical anomalies of the cystic duct only. The first (a male patient), underwent a hepatojejunostomy; he had a long and large-calibre cystic duct which was mistaken for the common hepatic duct (CHD). The second case (a female patient) had a spiral and very long cystic duct opening into the medial aspect of the CHD. Other common problems responsible for bile duct injuries are anomalies of the right hepatic duct (RHD) (e.g., low insertion on to the CHD), right anterior and posterior sections of the right hepatic duct, anomalies of the right hepatic artery and aberrant vessels coursing along the CBD.[9] All major extrahepatic CBD injuries in our series were recognized intra- operatively, whereas literature reports indicate that only 29% of the injuries are generally recognized intra-operatively.[9] The injuries of the bile duct may include partial tear, laceration, transection and even excision of a portion of the duct. These injuries are seen irrespective of the type of cholecystectomy and result in biliary stricture, which is undoubtedly the most serious complication following cholecystectomy. The severity of the complication depends on the type of injury, the delay in presentation, and on whether the patient requires a revision of an initial attempt at repair. Injuries identified and repaired at the time of the first operation afford good results.[3]

In our study, 38 patients’ (84.4%) operations were converted due to anatomical difficulties encountered intra-operatively, five due to the discovery of adhesions (11.2%) and two patients’ operations were converted due to common bile duct injuries (4.4%). It is clear from the literature that bile duct injuries occur even in the hands of experienced and competent surgeons but, obviously, inexperience increases the risks. A casual attitude towards a “simple” gallbladder (resection) may result in a catastrophe. A Swedish study[11] has shown that a surgeon is most likely to injure the bile duct when undertaking between his 25^th^ and 100^th^ operative cholangiogram. Acute inflammation around Calot's triangle makes the tissue friable and difficult to grasp. Dissection in such conditions leads to excessive blood being present. This, together with the distorted anatomy, increases the risk of bile duct injuries during LC.[10] On the other hand, extensive fibrosis around Calot's triangle in cases of chronically inflamed and fibrosed gallbladders may make them extremely difficult to dissect. The cystic duct and biliary tree system may be injured if the surgeon moves from the gallbladder down into the region of the bile duct to try and separate it and in doing so causes a diathermy injury to the bile duct, which can result in a leak. Occasionally, the CBD is dissected out and divided in the belief it is a cystic duct. In such cases, partial cholecystectomy is justified, as otherwise there remains a high risk of bile duct injuries. The probability of complications in the 1254 patients who underwent LC was significantly higher in those patients diagnosed with complicated gallstone disease.[12] Overzealous use of electrocautery near Calot's triangle and extensive dissection around the CBD may damage its axial blood flow, leading to ischemic damage to the duct and late stricture formation.[13] Excessive traction leading to tenting of the CBD is another factor predisposing to clipping and ligation of the bile duct, especially when performing an open cholecystectomy. An unnecessary attempt to demonstrate the junction of the cystic duct and the CBD can be potentially dangerous. Obesity and excessive fat in the porta hepatic area also poses technical difficulties and can predispose to bile duct injuries.[9]

Some authors have described the mechanism of “classic” laparoscopic injury in the presence of “normal” anatomy of the biliary tree; this pattern, occurs when the gallbladder is retracted superiorly. Surgeons believe they can see where the cystic duct is and dissect directly on to it, rather than dissecting on to the gallbladder. It is possible to follow what is believed to be the cystic duct down and then the CBD can be dissected out, clipped and then divided as the cystic duct.[13] Dissection proceeds upwards along the medial aspect of the CBD and the CHD until damage to the right hepatic artery results.

Other authors[14] have described a variation of this sequence of events, where faulty anterior and medial traction on the Hartmann pouch fails to open up Calot's triangle causing the cystic duct and the CHD to be mistaken. The CHD common hepatic duct junction is pulled up into the cystic duct and then clipped and divided. This can result in distal obstruction of the CBD and a fistula through the open cystic duct remnant. Clearly if the surgeon is aware of the existence of a short cystic duct then particular care needs to be taken when clipping it.

The factor responsible for the occurrence of such complications (as in our study) is the difficulty interpreting the two dimensional images seen in laparoscopic surgery. This is more likely to occur when no OC is performed. This mistaken interpretation and identification can be so compelling that the surgeon does not recognize an error has been made. Even when irregularities were identified intra-operatively, corrective action was rarely undertaken given the difficulty theatre staff may have in challenging firmly held assumptions of other staff.[15] Even when mistakes were identified post-operatively (e.g., in the presence of obvious jaundice) it has been found that appropriate feedback to relevant surgeons rarely occurred.[15] A review of 74 patients referred with bile duct injuries sustained during LC done at the Vanderbilt University Medical Center, Nashville, suggested that these injuries are frequently severe and are related to cautery and high clip ligation, and the level of injury was almost evenly divided between Bismuth type 3, 4, and 5 *vs* Bismuth type 1 and 2.[16,17]

Among the minor complications in our study, wound infection was the most common, followed by ileus, transient jaundice, and minor biliary leak. Minor biliary leaks were relatively unusual in our study (three of 22); two were due to the presence of an accessory duct and we were able to treat these cases conservatively; one case was due to the clips applied to the cystic duct slipping and this case had to be managed by percutaneous drainage and ERCP.

According to the literature, the leak may be minor,[18] arising from a small, accessory bile duct[19] and clinically insignificant. Such cases should be treated with percutaneous drainage. Injuries to the accessory bile duct are the most common cause of postoperative complications.[19] On the other hand, a major leak[3,18] due to injury to a main duct or a retained stone in the CBD[20] may result in biliary fistula, peritonitis, or biloma. Biliary fistula following LC is a common outcome in many studies; however, we had only one case in our series. Mostly, this complication results from improper application of clips or the clips slipping.[20] Use of diathermy to divide the cystic duct may cause charring of the tissue and failure of the clip to hold. ERCP helps in diagnosis and removes any doubts regarding possible major ductal injuries. The condition resolves spontaneously[20] provided there is no distal obstruction; the process may be hastened by the placement of a stent endoscopically.

In bile duct excision, a portion of the bile duct is lost and simple repair, as may be done in transection and laceration, is not possible.[21] This is the reason why both the cases with major extrahepatic biliary duct injures in this study underwent hepatojejunostomy. The chances of late stricture[21] are greater in bile duct transection than in bile duct laceration, as the axial vascular supply of the CBD is damaged in transection. Biliary reconstruction in the presence of peritonitis, combined vascular and bile duct injuries or injuries at or above the level of the biliary bifurcation were significant independent predictors of poor outcome.[21] In our study, all patients had excellent recovery and were discharged in a good condition within 10 days of surgery; however, long-term follow-up was not available. Strictures may develop early (within days or weeks) or may take years to develop and vary in both diameter and length.[22] Early strictures may develop due to intra-operative procedures such as clamping, ligation or clipping of the duct or thermal injury. Local infection may also result in both early and delayed stricture formation. Thermal injury and occult malignancy are important causes of delayed stricture formation. A thorough knowledge of the anatomy of the region, including possible anomalies, is important in preventing iatrogenic bile duct injuries. Both open cholecystectomy and LC are based on similar operative principles. Proper exposure and visualization, careful dissection, adequate haemostasis, careful placement of ligatures and clips, and division of structures only after proper identification are essential for safe cholecystectomy. Fundus-first cholecystectomy is well recognized as a safe technique during open cholecystectomy as well as during LC, because it minimizes the risk of injuries to the biliary structures at the Calot's triangle.[23]

Further specialised training to heighten awareness of the possible problems relating to the anatomy of the Calot's triangle is essential, not only for trainees but also for consultants engaged in this field. It is known that errors of judgement can be made relating to the few points within LC where complication-causing errors can occur, for example mistaking the CBD for the cystic duct or dissecting too close to the CHD.[15] An increased awareness of the possibility of such injuries could lead to a reduction in their occurrence and, further, the early recognition of such an injury (with the advantages of more training) would also lead to fewer injuries going unrecognised. Since injuries occurring at LC are frequently more severe and extend to a higher level than those that occur during open cholecystectomy (Strasberg E3–E5 injuries occur in 31% of LC *vs* 12% of open cholecystectomy), prevention should always be the aim.[24] Other authors have stressed the many steps that can be taken to prevent iatrogenic bile duct injuries.[9] For example, maximum cephalic fundal traction should be applied for better visualization of Calot's triangle; lateral and inferior traction on the Hartmann pouch can open up the angle between the cystic duct and the CHD and avoid problems of mistaken identification of relevant anatomy; Calot's triangle must be freed of fatty and areola tissue; dissection should start near the neck of the gallbladder (the cystic lymph node is an important landmark) and then proceed from the lateral to the medial direction, keeping close to the gallbladder; excessive and unnecessary dissection or use of electrocautery near the CBD must be avoided; cautery should be used at very low power setting in Calot's triangle[25] because electrocautery on tissues close to metal clips concentrates thermal energy and desiccates the tissue, making the clips less secure and thus predisposing to bleeding and biliary fistula formation. Any bleeding should be controlled only after accurate identification of its source and the neighbouring structures.

## CONCLUSION

Bile duct injuries are a rare complication of both open cholecystectomy and LC. It can have devastating effects, turning the individual into a “biliary cripple”. They mainly result from anatomical anomalies and errors of human judgment and are thus preventable to some extent. The costs are reduced and outcome improved if these injuries are diagnosed early (during operation or the early postoperative period). Adding the experience gained from open cholecystectomy on the one hand and the advantages of LC, in terms of visualization and magnification on the other, will help in reducing the incidence of such complications.
